# Stomatitis Materna

**Published:** 1872-11

**Authors:** I. P. Wilson


AMERICAN JOURNAL

OF

DENTAL SCIENCE.

Vol. VI. THIRD SERIES--NOVEMBER, 1872. No. 7.

### ARTICLE I.

### *Stomatitis Materna.*

BY DR. I. P. WILSON.

Read before the Illinois State Dental Society.

Stomatitis Materna, or as it is commonly termed "nursing sore mouth," is of frequent occurrence.

Writers differ as regards the cause of this disease. Nursing; climate; a particular condition of the blood; tubercular diathesis; epidemic influence, and an abnormal condition of the uterus, have all been given as causes for this peculiar affection.

These diversities of opinions lead us at once to the conclusion that the cause of this affection is not well understood.

All who have given the subject due consideration are agreed that it is not the nursing female alone that suffers from the disease, hence nursing cannot be the cause.

The probabilities are that climate has nothing to do with it. It is *more* probable that the habits, and manner of living peculiar to the people of different climes have their influence, and perhaps cause to a very great extent the disorder.

Writers assure us of its having prevailed in Holland, and that the disease is one of ancient date.

I agree that a disordered condition of the blood is always present with stomatitis, and that this disordered condition is the cause of the disease, yet there must be a cause for disordered blood, and that is the indirect cause of stomatitis materna. But more on this point before I close.

That the origin of this affection is from a tubercular diathesis, has been claimed by some writers; but to prove that this view of the subject is incorrect, we have only to call to mind the fact that the disease is not confined to that class of patients.

That certain influences cause it to prevail as an epidemic seems to be incredible, inasmuch as the disease is as common one year as another; provided, the habits and manner of living of the people of different countries are alike.

Prof. Pallen, of St. Louis, has claimed that this disease is due to an abnormal condition of the uterus. It is not strange that one should be led to this conclusion, for I doubt if stomatitis materna ever exists to any considerable extent, without an abnormal condition of that organ; not that this abnormal condition produces stomatitis materna, but it accompanies it, and both originate from the same cause.

We often see this disease, in its milder forms, in our dental practice, and it was here that my observation commenced, and, for a number of years I have given the subject careful consideration, and am now of the decided opinion that the disease is due to a low condition of the system—a lack of vitality—*positive starvation.*

The human body is composed of fifteen different elements. These elements are undergoing a continual change—growth and decay.

The elements from which these tissues were made, and these bodies formed, were derived from the food we eat and drink. If our ailment does not contain these elements, or if the supply is too meagre to meet the demand, healthful, vigorous tissues will not be formed and the entire system must suffer.

Milk and eggs are said to be the only articles of food that furnish all the elements found in our system. This is obvious from the fact that the child's muscles and bones are formed from the nourishment it receives from its mother's breast; and the little chick receives from the egg all the elements necessary for its formation. Deprive milk of its lime salts, and what will become of the bony structure of the child; and likewise, remove the phosphates from the egg, and where is the framework of the little chick?

Withdraw bone producing material from our food, and the whole osseous system must suffer.

The bones contain from 48 to 59 per cent of the phosphate of lime, and the enamel of the teeth contains from 81 to 88 per cent. Remove these elements from our food and the system becomes reduced, and if it be a mother with an infant at her breast, her system will be reduced to extreme poverty.

Instead of the richly flowing current in her veins, infusing life, and health, and sustenance to the surrounding tissues of the body, there flows the poverty-stricken stream, unable to impart repairing material to the broken down tissues, and at such a time, the disease in question will manifest itself.

In extreme cases of stomatitis materna, the affection is not confined alone to inflammation of the mucous membrane of the mouth, but the fauces, and sometimes the whole alimentary canal are affected. As I have stated above, Prof. Pallen, of St. Louis, informs us, that he always finds the uterus in an abnormal condition, and I have no doubt but this is strictly true, and that the abnormal condition referred to, is precisely the same as found in the mouth and fauces; that the two make their appearance simultaneously, and have the same origin. In fact there can be but little doubt, but all the mucous membranes are more or less affected at the same time.

Females, during gestation and lactation, suffer more frequently from this affection than any other class, because of the immense drain made upon their systems during those periods.

The manner in which our food is prepared furnishes us with a scanty supply of the lime salts, so that the non-pregnant female and the male sex too, have only a limited supply of the phosphates, and if it is true that these classes receive barely enough of the phosphates to keep their systems in repair, how shall the mother, during gestation and lactation, meet the demands made upon her system.

It is nature's provision that the helpless infant shall draw its life from the veins of its mother. It has no other means of support; God has furnished it its food ready for use, because it is helpless and dependent and cannot provide for itself. Not so with man's food. True, our bountiful Benefactor gives man the golden harvest, but He does not prepare the flour and the loaf. It is for man to do this. And as he grinds the wheat and bolts the flour, he removes from it those life-supporting elements required to sustain the bony structure. Bolted flour is almost entirely destitute of the lime salts, while the unbolted flour contains about twelve per cent. of the phosphates.

It is said that paring a potato removes from it a greater portion of the phosphates, as these salts are found more abundantly near the surface.

Stomatitis is not confined to one sex; both are liable to this disease. Pregnant and nursing females are much more frequently affected; non-pregnant females next, and the male sex least. The first, because she furnishes material for the growth and development of her child. The non-pregnant female is more liable than the male, because she lives an in-door life. The male sex least, because he is more frequently in the open air, and in the sun. It is just as essential that we should have the fresh air and the warm rays of the sun, as it is for the plants in our window.

I have said that the male sex may be affected with this disease. I have seen such cases, when, if my patient had been a mother, I should have pronounced the disease "nursing sore mouth." But these are exceptional cases. The mother is usually the sufferer. See her trembling and emaciated form, with her healthful child full of life and vigor, nestling in her bosom. She, who a few years since, had an elastic step, a sparkling eye, and a rosy cheek, and whose spirits were buoyant, and whose merry bursts of laughter gladdened the hearts of those around her. See her, and ask why all this change.

The infant in her arms has grown strong and vigorous, as her life-blood has gently flown into its veins to enrich them, but to impoverish her own.

At such a time the teeth very clearly indicate the starved condition of the system. They become extremely sensitive; there is a softening of the tooth structure, and they decay with great rapidity.

The mother, then, should use bread made from the unbolted flour, and should take exercise in the open air and in the sun. I have seen this disease cured in a very brief period of time, by a close attention to diet and exercise, as I have above directed.

Finally, I would say, let us keep the "house we live in" in repair. It is our own domicile—our earthly home. Let us provide for it with fostering care; let us cherish and *love* it.

---

